# Resounding Meaning: A PERMA Wellbeing Profile of Classical Musicians

**DOI:** 10.3389/fpsyg.2018.01895

**Published:** 2018-11-06

**Authors:** Sara Ascenso, Rosie Perkins, Aaron Williamon

**Affiliations:** ^1^Centre for Performance Science, Royal College of Music, London, United Kingdom; ^2^Faculty of Medicine, Imperial College, London, United Kingdom

**Keywords:** PERMA, wellbeing, classical musicians, positive psychology, meaning

## Abstract

While music has been linked with enhanced wellbeing across a wide variety of contexts, the professional pursuit of a music career is frequently associated with poor psychological health. Most research has focused on assessing negative functioning, and to date, few studies have attempted to profile musicians’ wellbeing using a positive framework. This study aimed to generate a profile that represents indicators of optimal functioning among classical musicians. The PERMA model, which reconciles hedonic and eudaimonic wellbeing, was adopted and its five elements assessed with a sample of professional classical musicians: Positive Emotion, Engagement, Relationships, Meaning and Accomplishment. 601 participants (298 women, 303 men) engaged in careers as orchestral (*n* = 236), solo (*n* = 158), chamber (*n* = 112), and choral musicians (*n* = 36), as well as composers (*n* = 30) and conductors (*n* = 29), answered the PERMA-Profiler, a self-report questionnaire built to assess the five components of PERMA. Results point to high scores across all dimensions, with Meaning emerging as the highest rated dimension. Musicians scored significantly higher than general population indicators on Positive Emotion, Relationships and Meaning. When wellbeing is assessed as positive functioning and not the absence of illbeing, musicians show promising profiles. The reconciliation between these findings and the previous body of research pointing to the music profession as highly challenging for healthy psychological functioning is discussed.

## Introduction

Music is a pursuit with tremendous personal, social and cultural significance and is widely seen as a privileged enhancer of wellbeing across a variety of contexts. *Listening* to music in everyday life has been related to positive emotions (e.g., Laukka, 2007; Gabrielsson, 2011), lower levels of reported stress and enhanced emotional regulation (Västfjäll et al., 2012). Studies with functional magnetic resonance imaging (fMRI) have also provided insight into why listening to music is one of the most rewarding of human experiences, through clarifying its link with enhanced functional and effective connectivity between brain regions mediating reward, autonomic, and cognitive processing (e.g., Menon and Levitin, 2005). *Making* music has also been associated with wellbeing. Community initiatives in particular are progressively gaining support as a prolific forum for the enhancement of positive functioning. The specific case of community singing has been the focus of much attention as a mediator for a positive effect on self-reported mood (Valentine and Evans, 2001), heightened perception of quality of life (Clift, 2012), positive relationships, meaning (Clift and Hancox, 2001; Davidson, 2004) and engagement (Davidson, 2011). The impact of singing at a biological level is also well documented. For example, a study with members of a mixed amateur choir found improved immune function (as measured by secretory immunoglobulin A [S-IgA] and cortisol) and affect (as measured by the Positive and Negative Affect Schedule – PANAS) after one session of singing (Kreutz et al., 2004). Community drumming also features in recent studies as particularly useful for wellbeing promotion with vulnerable groups such as older populations (Perkins and Williamon, 2014) as well as with mental health service users, where recent work highlighted positive changes at both psychological and biological levels after short interventions of six or ten sessions of group drumming (Fancourt et al., 2016; Perkins et al., 2016; Ascenso et al., 2018). In academic settings, music-engagement activities emerge as significant contributors to wellbeing through both the enhancement of positive emotions and as facilitators of optimal functioning through potentiating creativity, self-efficacy (Boyce-Tillman, 2000) and social skills (Schellenberg et al., 2015). Notably, this has included populations of students with special needs, such as syndromes on the autistic spectrum (Ockelford, 2012). Within clinical settings, music therapy has gained attention as a valuable addition to established treatment practices in major conditions such as depression (Choi et al., 2008), substance abuse (Aldridge and Fachner, 2010) and delinquency (Chen et al., 2016). Furthermore, both listening to and making music have been successfully used for neurologic rehabilitation within varied conditions: stroke-induced motor dysfunction (Altenmuller et al., 2009), Parkinson’s disease (Arias and Cudeiro, 2008) and aphasia (Belin et al., 1996). Additionally, music interventions targeted at behavioral symptoms of neurologic clinical profiles, like Alzheimer’s disease, have also proven effective. Groene (1993), for example, highlighted an increase in engagement, through reducing wandering behavior after a music-attention intervention. Strong evidence supporting the efficacy of music for pain relief has also emerged (Mitchell and MacDonald, 2012), and positive effects of music, in particular linked with communication, have been consistently found in interventions with chronically ill patients, as measured by qualitative inquiry (Pothoulaki et al., 2012). Music is also offering clinicians scientifically based options for cost reduction with medication targeting anxiety control in the context of surgical procedures (Bringman et al., 2009; Spintge, 2012).

In contrast with such positive outcomes, musical activity at a professional level has been widely equated as a threat to wellbeing through several mediators. Firstly, musicians consistently report high rates of performance-related pain and physical discomfort (Ackermann et al., 2012; Cruder et al., 2017) and of non-specific musculoskeletal disorders (Fishbein et al., 1988; Watson, 2009). High incidence of hearing problems has also been identified (Kähäri et al., 2003; Hagberg et al., 2005; Hasson et al., 2009; Schink et al., 2014). Both work-related physical injury and hearing disturbances have been found to play a significant influence on psychological health (Dersh et al., 2002; Bair et al., 2008; Krog et al., 2010).

Performance anxiety has also received major attention as a factor for ill health among musicians. This is a condition that has been reported to affect individuals in a range of endeavors, from public speaking (Merritt et al., 2001; Blöte et al., 2009), test-taking (Elliot and McGregor, 1999), sports (Hall and Kerr, 1998; Hanton et al., 2002) and other performing arts (Wilson, 2002) such as dance (Walker and Nordin-Bates, 2010) and acting (Steptoe et al., 1995). Performance anxiety has not been classified in the Diagnostic and Statistical Manual of Mental Disorders (DSM) outside of a differential diagnosis for social phobia. For the case of Music Performance Anxiety (MPA) (Kenny and Osborne, 2006), it has been argued that it may or may not impair performance quality (Barlow, 2002), and there is also debate as to how much it is related to the degree of preparation and to whether the definition should include mention of this (Kenny, 2011). Nevertheless, all definitions are unanimous in considering the triad of interactive, yet partially independent, symptoms – cognitive, somatic and behavioral (Craske and Craig, 1984) – that sustain a persisting distressful experience in performance situations (Kenny, 2011). Most MPA research has been developed with orchestral musicians and is still limited to cross-sectional studies. Furthermore, the most comprehensive and large-scale findings are considerably dated. Prevalence percentages vary widely. Studies by Fishbein et al. (1988) and Lockwood (1989), based on the survey from the International Conference of Symphony and Opera Musicians (ICSOM), reported that 24% of participants suffered from MPA. Higher percentages have been found by van Kemenade et al. (1995) at 58% and James (1998) at 70%. Brugués (2011) argues that this variability can be attributed to the different operationalization of terms used in each study. Fishbein et al. (1988) and Lockwood used ‘stage fright,’ while van Kemenade et al. (1995) inquired about ‘performance anxiety,’ and James equated it as an experience of anxiety severe enough to interfere with performance.

Studies with conservatoire students report significantly higher performance anxiety than those with professionals (Steptoe and Fidler, 1987; Kenny, 2011). The major self-reported causes of MPA in tertiary-level students have been identified as: inadequate preparation, pressure from self, general lack of confidence, difficult repertoire, and excessive physical arousal (Kenny, 2011). Women are two to three times more likely to experience anxiety than men (American Psychiatric Association, 1994; Lewinsohn et al., 1998), and this relationship holds for music performance anxiety as well (Sinden, 1999; Huston, 2001; Osborne and Franklin, 2002). Furthermore, solo performers have shown higher MPA scores than ensemble members (Cox and Kenardy, 1993). Overall, despite mentioning the same construct, MPA is operationalized differently across studies, and therefore, the possibility for comparisons is generally limited.

Adding to physical injury and performance anxiety, musicians have been associated with high levels of general psychological illbeing. For example, Voltmer et al. (2012) highlighted that, when compared with general population samples, opera and orchestral musicians in Germany showed higher prevalence of mental distress as measured by the Short Form-12 general health questionnaire (SF-12). Interestingly, in relation to physicians and aircraft manufacturers, however, no differences were found. It has also been suggested that creativity, seen as a general prerequisite for artistic endeavors, is associated with increased risk of affective disorders (Akiskal et al., 2005; Mula and Trimble, 2009). In line with this, patients with schizophrenia or bipolar disorder have been found to be overrepresented in creative occupations (such as research and artistic professions) (Kyaga et al., 2011; Kyaga et al., 2013). A recent study in Norway focusing on psychological illbeing among musicians, particularly anxiety and depression, found that musicians scored higher for both when compared with a general workforce sample (Vaag et al., 2015). The highest prevalence rates came from soloists, vocalists, keyboard instrument players and string players. Women were linked to a higher prevalence of psychological distress than men, in line with previous research with general samples (Rosenfield and Mouzon, 2013). When bringing in other professional groups for comparison, musicians differed substantially from managers, technicians and academic professionals, scoring higher than all. A previous study with participants from symphony orchestras in Denmark (Holst et al., 2012) had also highlighted that compared with a sample from general population, musicians reported higher emotional demands, lower job satisfaction, lower decision latitude, lower social support and lower sense of community. The same study found a higher degree of perceived stress among female musicians.

Two recent cross-sectional studies also suggested a propensity for disorder. Ackermann et al. (2014) investigated a sample of professional orchestral musicians in Australia and found symptoms of social phobia (33%), depression (32%) and PTSD (22%). Barbar et al. (2014) found a high rate of disorder indicators and susceptibility to illbeing in Brazilian musicians: a 13% prevalence of moderate or severe degree of symptoms of general anxiety, 19% prevalence of social anxiety symptoms and 20% prevalence of depression symptoms. Professional musicians have also been associated with high mental fatigue (Steptoe, 1989) and boredom (Parasuraman and Yasmin, 2000). Accounts by Sternbach (1995) have shed light into specific job-related stressors for musicians that may help explain the negative picture these studies have provided, namely: disruption in family life; employment insecurity and difficulties in career development; the roller-coaster of underload/overload of work already highlighted by Cooper and Wills (1989) and problematic aspects of person-environment fit. A study with Finnish orchestras, however, found rates of extreme job satisfaction of around 90%, a significantly higher result when compared with other professions, namely: clerical workers (40–70%), human relation workers (70%) and industrial workers (50–60%) (Kivimäki and Jokinen, 1994). Despite still reporting high levels of job-related strain, musicians’ satisfaction with work did not seem to be affected. The authors also report higher perceived skill-variety (assessed in the study as great possibilities to use personal knowledge and skills), and suggest job satisfaction levels might be explained by orchestral work offering a platform for greater self-realization when comparing with the other occupations investigated in the study. Perceived stress levels were also higher when compared with scores from clerical and industrial professions and comparable with results from human relation workers.

As is clear from the diversity of research reviewed, listening to and making music are strongly linked with wellbeing, yet at the same time, professional musicians themselves are exposed to specific, performance-related risks that may threaten their healthy functioning. One of the issues that arises from both sets of studies is the conceptual blurriness and lack of convergence in the underlying theoretical grounds for the wellbeing construct itself. In the cases where wellbeing is defined, several definitions concur across studies (and even within the same study), representing oftentimes different phenomena altogether. This hinders the possibility for comparison. Additionally, despite enabling awareness of key factors within professional musicians′ wellbeing (by definition an inherently positive construct), the research base within this field has been dedicated almost exclusively to profiling ill-functioning. While a few studies recognize the relevance of a positive health approach, the assessment of wellbeing remains limited to the absence of negative indicators (e.g., performance anxiety, distress). The field would largely benefit from both a greater clarity of its theoretical grounds and a more comprehensive approach to measurement. Before introducing our study, it will be useful to turn to the various definitions of wellbeing that have dominated the literature, to help operationalize the construct.

Broadly, wellbeing refers to the experience of feeling good and functioning well. Since the start of psychology as a scientific discipline, the focus of mental health and wellbeing research and practice was placed on the treatment and prevention of conditions such as depression and anxiety. It was generally assumed that wellbeing would emerge when pathology was absent. However, within early accounts of research in this field, there was a major finding that was key to laying the groundwork for an altogether new concept of wellbeing. Bradburn (1969) showed that pleasant and unpleasant affects are independent and have different correlates, not simply standing as opposites to one another or different ends of the same continuum. The implications were straightforward as psychologists realized that positive and negative affect should be studied separately and that successful attempts to eliminate negative states do not necessarily translate in an increase in positive functioning. This had also been preceded by the key work of renowned social psychologist Marie Jahoda, who very timely suggested that “the absence of mental illness is not a sufficient indicator of mental health” (Jahoda, 1958, p. 15), pioneering the concept of positive mental health. This work paved the way for what has now become a solid field of research. Following a progressive shift of paradigm, research has led to new definitions of mental health. The construct of ‘being psychologically well’ refers now to a state that is qualitatively different from the absence of mental illness and, as Seligman (2008) points out, stands as a quantifiable and predictive entity, defined by a combination of excellent status on biological, subjective, and functional measures. This shift of paradigm deviates markedly from the focus on merely minimizing harm that has dominated much of the field for decades. As a result, an outpouring of research on wellbeing and efforts to define, assess and plan interventions focusing on empowerment of strengths has emerged (Bolier et al., 2013). It is also becoming clearer that the impact of high wellbeing goes beyond optimized psychological and physical functioning in the present but stands as predictor of future positive outcomes such as productivity and job satisfaction, relationship stability, physical health and longevity (Lyubomirsky et al., 2005; Fredrickson, 2009).

The field of wellbeing research has generally encompassed two perspectives: the hedonic and the eudaimonic. The first equates positive human experience as centered on positive affect. In this context, wellbeing is generally defined as the optimal balance between positive and negative affect, along with perceived satisfaction with one’s life. Proponents of this view have included Bradburn (1969), Watson et al. (1988), and Diener et al. (1999). The eudaimonic approach, in turn, has been centered on virtuous action and self-realization. Wellbeing is equated as the degree to which a person is fully functioning and actualizing one’s potential (Waterman, 1993; Ryan and Deci, 2001). Whereas the hedonic perspective does not specify a single formula for wellbeing, placing centrality on the subjective construction by the individual, the eudaimonic perspective adopts a more theory-guided approach. It argues that focusing on affect and life satisfaction alone neglects important aspects of functioning and that the experience of wellbeing cannot be represented exclusively by the individual’s evaluative perceptions. In recent years, research has highlighted the relevance of re-equating wellbeing as a combination of both hedonic and eudaimonic components, bringing the two approaches steadily towards convergence (Samman, 2007).

One attempt to reconcile hedonic and eudaimonic traditions is *The Wellbeing Theory* or PERMA model, put forth by Seligman (2011), which proposes five building-blocks for wellbeing: Positive Emotion, Engagement, Relationships, Meaning and Accomplishment. In order to qualify as an element of wellbeing, Seligman argues that each component must have the following three properties: (1) it contributes to wellbeing, (2) it is pursued for its own sake, and (3) it is defined and measured independently from the other components (Seligman, 2011).

*Positive Emotion* refers to the affective component or feeling well, in combination with a positive appraisal (Seligman, 2011). Numerous reviews support the value of positive emotion across a range of life outcomes such as physical health, longevity, psychological stability, cognitive performance and work productivity (Lyubomirsky et al., 2005; Howell et al., 2007; Huppert, 2009).

*Engagement* refers to a deep psychological connection to a particular activity, organization or cause. It is the psychological state in which individuals are absorbed in a task (Forgeard et al., 2011) implying interest, intense involvement, effort and immersion. Research on engagement has occurred across several relatively disparate domains. Measures have focused primarily on flow, equated as an extreme level of psychological engagement that involves intense concentration, absorption and focus (Csikszentmihalyi and Csikszentmihalyi, 1988). Irrespective of the type of task, it occurs only when the individual moves beyond his or her average experience of challenge and there is complete investment. These experiences, described as autotelic, bring high intrinsic reward and motivation to return to them. In a state of flow, action and thought become merged and awareness of feeling is usually absent (Csikszentmihalyi, 1991). Thus, as Seligman (2011) points out, while the state of positive emotion is a present state, the subjective state for engagement is retrospective. Levels of flow have been associated with increasing motivation and creativity in both work and leisure contexts (Csikszentmihalyi and LeFevre, 1989).

The element of *Relationships* refers to the perception of both quantity and quality of social connections. It implies the belief that one is cared for, loved and valued (Seligman, 2011). Social relationships have been considered the most central element of wellbeing (Berscheid and Reis, 1998), and their impact on optimized functioning has been extensively studied. A recent review by Tay et al. (2013) of studies in the past 10 years found over 18,000 articles published on social relationships and health. Social support has been linked to less psychopathology, better physical health, lower mortality risk, health-promoting behaviors, chronic illness self-management and decreased suicidal tendencies (Taylor, 2011; Tay et al., 2013). Sub-domains include social ties (number of persons in social sphere), social networks (number of ties and quality of those ties), received support (objective perspective of resources), perceived support (subjective perspective of resources), satisfaction with support, and giving support to others (Taga, 2006).

*Meaning*, closely linked to purpose, has been defined as the “ontological significance of life from the point of view of the individual” (Crumbaugh and Maholick, 1964, p. 201), or the feeling of belonging and serving something larger than the self (Seligman, 2011). Meaning provides a sense that one’s life matters. It has been associated with better physical health, reduced mortality risk, and higher life satisfaction (Ryff et al., 2004; Boyle et al., 2009; Steger, 2012).

The final component of PERMA refers to *Accomplishment* or success and mastery (Forgeard et al., 2011). Accomplishment encompasses both external indicators and internal goals. Although accomplishment can be defined in objective terms, this model places centrality on the *perception* of accomplishment (Seligman, 2011).

For professional musicians, studies of the experience of wellbeing have not escaped the pathology-orientated tendency that has been dominant within the broader field of psychology. Research leading to wellbeing interventions has been scarce and typically focused on addressing music performance anxiety, physical injury and general debilitating factors found within the music profession. Following positive psychology’s appeal to re-direct efforts to more than the alleviation of symptoms, and acknowledging the need to go beyond MPA as the central indicator of musicians’ psychological functioning, it seems timely to re-examine musicians’ wellbeing through a new lens, focusing on positive elements and incorporating wellbeing’s multidimensionality. One of the most widely disseminated definitions of mental health is that proposed by the World Health Organization (WHO): “a state of wellbeing in which every individual realizes his or her own potential, can cope with the normal stresses of life, can work productively and fruitfully, and is able to make a contribution to her or his community” (World Health Organization [WHO], 2005, p. 1). The positive essence is also stressed in WHO’s definition of general health as contained in its constitution: “Health is a state of complete physical, mental and social wellbeing and not merely the absence of disease or infirmity” (World Health Organization [WHO], 2005, p. 1). Indeed, if wellbeing is more than the absence of illbeing, it seems that, to date, musicians’ wellbeing has not been comprehensively targeted. The assessment of all components of PERMA aiming for an idiosyncratic description of musicians seems a valuable step for paving the way toward a better understanding of how this professional group experiences wellbeing as truly the presence of positive functioning. With this in mind, we aimed to generate a large-scale profile of the PERMA components among professional musicians. Our previous qualitative investigation with the same framework (Ascenso et al., 2017) assessed the experience of six high-profile professional musicians on the five components of PERMA through interviews and diary record-keeping. Interpretative Phenomenological Analysis (IPA) highlighted the centrality of eudaimonic components and allowed for clarification on the ingredients contributing to each component of PERMA in a musician’s typical routine. Positive Emotion emerged highly related to musical moments, while varying repertoire and experiencing different ensembles appeared as central sources of engagement. Meaning emerged as linked to the shared nature intrinsic to music-making, and a sense of accomplishment was built primarily on internal goals and a perception of oneness in performance with others. Relationships assumed a leading role in musicians’ self-reports of positive functioning. A clear sense of identity was found to be perceived as an over-arching sustainer of wellbeing, and the transition to professional life as the most challenging phase (Ascenso et al., 2017). Following-up from this study, and in an attempt to contribute to a profile of musicians’ wellbeing that is truly a *wellbeing* profile, we were interested in exploring quantitative tendencies on PERMA scores across a large sample, allowing for comparisons with general population indicators.

## Materials and Methods

### Participants

A convenience sample of professional classical musicians was recruited via email through international music ensembles, educational institutions and performance companies and festivals, well positioned in the classical music scene: orchestras, choirs, opera houses, chamber ensembles and conservatoires. Forty-five organizations across 41 countries collaborated. Freelance musicians also took part, having learned of the project through their former educational institutions, agents or previous ensembles. A central criterion for inclusion in the study was that musicians should be actively involved in performance-based music-making as their main source of income. Musicians working as instrumental teachers or with parallel careers were included as long as their cumulative performance or composition activities represented the most significant part of a typical working week. Additionally, all participants were fluent in English and over 18 years old.

A total of 700 musicians volunteered to participate, and 601 (86%) returned complete data sets (298 women, 303 men). Age was indicated in the following bands: 18–24 (*n* = 73), 25–39 (*n* = 280), 40–54 (*n* = 161), 55–64 (*n* = 70), over 65 (*n* = 17). Six types of professional musical activity were represented: orchestral (*n* = 236), solo (*n* = 158), chamber (*n* = 112) and choral musicians (*n* = 36), as well as composers (*n* = 30) and conductors (*n* = 29). The majority had more than 5 years of professional experience (*n* = 456, 75.9%); only 2.3% had worked professionally for less than a year, and 21.8% of participants had between one and 5 years of experience. The United States and the United Kingdom were the most represented countries for both nationality (23 and 22%, respectively) and place of work (17 and 11%, respectively).

This study was granted ethical approval by the Conservatoires UK Research Ethics Committee and was conducted according to ethical guidelines of the British Psychological Society. Informed consent was obtained from all respondents, and no payment was given in exchange for participation.

### Procedure

Respondents completed the PERMA-Profiler (Butler, 2011, unpublished; Butler and Kern, 2016), designed to assess multi-dimensional wellbeing along the five components of the PERMA model (Seligman, 2011). This measure was built as a 15-item survey with each item scored on a likert-type scale from 0 to 10 (where higher scores indicate greater wellbeing). Three items assess each PERMA construct, and composite scores are averaged across the three items per construct. A general item, with the same 10-point scale, is included as an overall evaluation of happiness. *Overall Wellbeing* is the average of the 15 main PERMA items plus the overall happiness item.

The measure includes additional items assessing negative functioning (three items for negative affect, forming the *Negative Emotion* subscale, and a single item for loneliness) and the perception of physical health (three items). This study used the primary PERMA measure with the 15 main items as its core tool (NB: at the time of the study, the original scale did not include the assessment of satisfaction with physical health). The *Negative Emotion* subscale was also included, for two reasons. As Butler and Kern (2016) point out, besides providing useful information through acknowledging the importance of considering both positive and negative elements of functioning, these items also act as filler items that disrupt response tendencies. The PERMA-Profiler has demonstrated acceptable internal reliability and good overall fit in studies including over 15,000 participants worldwide (Butler and Kern, 2016). In the present study, the measure was administered in both paper-based and online versions. The online version was built and delivered using the platform Survey Monkey.

### Data Analysis

Descriptive statistics were calculated for all PERMA factors, *Overall Wellbeing* and *Negative Emotion*. Pearson correlations were calculated between wellbeing elements, and independent samples *t*-tests using summary values were run to compare musicians’ scores with general population indicators. Factorial ANOVAs including Sex, Age, Years of Experience, Type of Activity and the two-way interactions between Sex and each of the other three factors were run for each PERMA component and for *Overall Wellbeing* and *Negative Emotion*. Cohen’s *d* and Cohen’s *f* were used to estimate effect sizes for *t*-tests and ANOVAs, respectively. Three-way interactions or two-way interactions between Age, Years of Experience and Type of Activity were not considered because these would have resulted in categories with no data or very small sample sizes (*n* < 5). Although Age and Years of Experience are expected not to be completely independent, the correlation between the two variables was only moderate (Spearman’s rank correlation rho = 0.468) which justified including both in the same analysis. All PERMA factors showed left-skewed distributions that did not deviate severely from normal. Due to the large sample sizes, no transformations were performed for *t*-tests or correlation analyses but greater precaution was taken for the factorial ANOVAs to meet all the assumptions. A quadratic transformation was applied to all PERMA factors and a cube transformation was applied to *Overall Wellbeing*. For *Negative Emotion*, no transformation was shown to improve the distribution of the residuals, which were already very close to normal, so the ANOVA was performed without transforming the data.

Correlations and independent samples *t*-tests were performed using IBM SPSS Statistics for Windows, version 24.0 (IBM Corp., Armonk, NY, United States). The factorial ANOVAs were conducted using R Statistical Software Version 1.1.453 (R Core Team, 2013).

## Results

Table 1 shows descriptive statistics for each PERMA sub-scale as well as Cronbach’s Alpha reliability indicators. All PERMA component scores were, on average, above the mid-point of the scale, and the mean for *Overall Wellbeing* was *M* = 7.34 (*SD* = 1.68). Component scores ranged from *Positive Emotion* as lowest (*M* = 7.06, *SD* = 1.50) to *Meaning* as highest (*M* = 7.64, *SD* = 1.58). Reliability indicators were good or acceptable for all subscales following Nunnally (1978) criteria, with the exception of the *Engagement* scale with a lower α (0.51), indicating the least reliable sub-scale, in line with findings from the scale’s validation sample (Butler and Kern, 2016).

**Table 1:** Means, confidence intervals, standard deviations and Cronbach’s Alpha (α) reliability indicators for each PERMA component, Overall Wellbeing and Negative Emotion.

Component	*N*	Mean	*SD*	95% Confidence Interval for Mean		α
				Lower	Upper	
Positive Emotion	601	7.06	1.50	6.94	7.18	0.85
Engagement	601	7.33	1.34	7.22	7.44	0.51
Relationships	601	7.25	1.70	7.11	7.38	0.72
Meaning	601	7.64	1.58	7.52	7.77	0.80
Accomplishment	601	7.32	1.38	7.21	7.43	0.71
Overall Wellbeing	601	7.34	1.68	7.24	7.44	0.88
Negative Emotion	601	4.04	1.76	3.89	4.18	0.69

*Scales range from 0 to 10, where 0 = lowest and 10 = highest.*

Table 2 provides correlations between the PERMA factors for the overall sample. As in the original study (Butler and Kern, 2016), all PERMA factors were significantly positively correlated with each other, and correlations were mostly moderate. For instance, as participants reported greater *Positive Emotion*, they also tended to report higher levels of *Engagement* (*r* = 0.516), satisfaction with *Relationships* (*r* = 0.561), *Meaning* (*r* = 0.683) and *Accomplishment* (*r* = 0.640).

**Table 2:** Correlations among the five PERMA components.

	*P*	*E*	*R*	*M*	*A*
*P*	1				
*E*	0.516^∗∗^	1			
*R*	0.561^∗∗^	0.342^∗∗^	1		
*M*	0.683^∗∗^	0.519^∗∗^	0.518^∗∗^	1	
*A*	0.640^∗∗^	0.427^∗∗^	0.454^∗∗^	0.659^∗∗^	1

*^∗∗^p < 0.01 P, Positive Emotion; E, Engagement; R, Relationships; M, Meaning; A, Accomplishment.*

There were no significant effects of Sex, Age, Years of Experience, Type of Activity as well as no significant interactions between factors for *Engagement* or *Accomplishment* (all *p*-values ≥ 0.1237). The interaction effects between Sex and Type of Activity were significant for *Positive Emotion* (*F* = 3.6723, df = 5, *p* = 0.0028, *f* = 0.173), *Relationships* (*F* = 3.3651, df = 5, *p* = 0.0052, *f* = 0.169), *Meaning* (*F* = 3.1083, df = 5, *p* = 0.0089, *f* = 0.163) and *Overall Wellbeing* (*F* = 3.9691, df = 5, *p* = 0.0015, *f* = 0.179). Additionally, the interaction between Sex and Years of Experience had a significant effect for *Positive Emotion* (*F* = 3.7542, df = 2, *p* = 0.0240, *f* = 0.113) and *Overall Wellbeing* (*F* = 3.4308, df = 2, *p* = 0.0330, *f* = 0.108), whilst Age was a significant factor for *Meaning* (*F* = 4.3669, df = 4, *p* = 0.0017, *f* = 0.173) and *Negative Emotion* (*F* = 2.9345, df = 4, *p* = 0.0202, *f* = 0.167). Years of Experience was a marginally significant factor for scores in *Negative Emotion* (*F* = 3.1290, df = 2, *p* = 0.0445, *f* = 0.103) and the assumption of homogeneity of residuals was not met for this variable, so these comparisons must be interpreted with caution.

**FIGURE 1:**
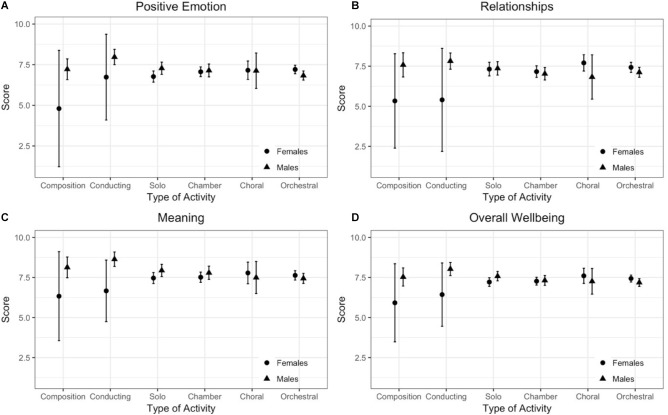
Mean scores with 95% CI for male and female professional musicians, per area of musical activity, for the wellbeing scales where an interaction effect between Sex and Type of Activity was significant, as determined by a factorial ANOVA. **(A)** Positive Emotion, **(B)** Relationships, **(C)** Meaning, **(D)** Overall Wellbeing.

The interaction between Sex and Type of Activity followed a very similar pattern across the three PERMA factors and *Overall Wellbeing* (Figure 1): male conductors showed greater scores than musicians from other areas of activity, particularly for *Meaning* and for *Overall Wellbeing.* There were no obvious differences on average scores between men and women for all types of musical activity, with the exception of some indication of possible gender differences among conductors and composers, where women showed lower averages compared with men and, generally, compared with both men and women of other types of activity. The confidence intervals for women’s means in these two groups were particularly high due to the very small sample sizes (5 female composers and 5 female conductors), and therefore the interpretation of this pattern is extremely cautious. There was also some indication of the opposite trend amongst orchestral players, with slightly higher average scores for women than those of men across all significant PERMA elements and *Overall Wellbeing*.

**FIGURE 2:**
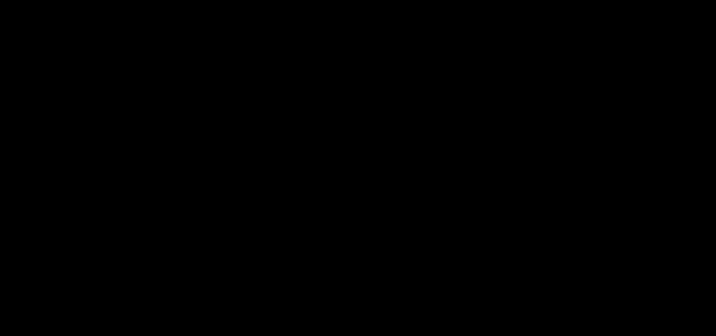
Mean scores with 95% CI for male and female professional musicians, per category of Years of Experience, for each of the wellbeing scales where an interaction effect between Sex and Years of Experience was significant as determined by a factorial ANOVA. **(A)** Positive Emotion, **(B)** Overall Wellbeing.

The interaction between Sex and Years of Experience suggests that, for both *Positive Emotion* and *Overall Wellbeing*, women score on average lower than men earlier in the career (for categories with experience under 1 year and between 1 to 5 years), with scores between sexes remaining similar for those with over 5 years of professional experience. For men, on average, scores are higher earlier in the career (under 1 year of experience) whilst scores among women are higher later in the career (over 5 years of experience) (Figure 2).

**FIGURE 3:**
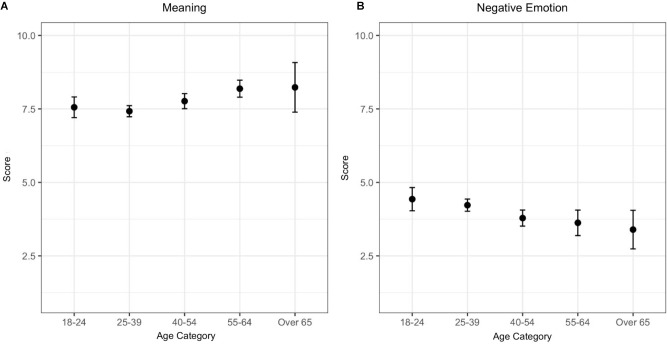
Mean scores with 95% CI across age categories for professional musicians. **(A)** Meaning and **(B)** Negative Emotion.

When looking at the effects of Age, there is a tendency across the five age categories for an increase in *Meaning* and a decrease in *Negative Emotion* (Figure 3).

In terms of the effect of Years of Experience on *Negative Emotion*, musicians with between 1 and 5 years of experience showed higher average scores for *Negative Emotion* (*M* = 4.46, *SD* = 1.64), followed by those with over 5 Years of Experience (*M* = 3.94, *SD* = 1.79) with those with less than 1 year experience showing the lowest average scores (*M* = 3.36, *SD* = 0.866). When controlling for the effect of Age, which showed a greater effect size, only the comparison between less than 1 year of professional experience and between 1 to 5 years was marginally significant (*p* = 0.0438).

Table 3 shows comparisons between musicians’ results and general population results as published by Butler and Kern (2016). The comparison sample (*N* = 31,966) represents the combination of 11 sub-samples from the scale’s validation studies with adults from age 18 to over 65, from across a very wide variety of occupations and professional activities. Despite including participants from all over the world, the USA accounts for approximately 60% of the sample.

**Table 3:** Independent samples *t*-test results comparing mean scores of musician and general population groups for each PERMA component, Overall Wellbeing and Negative Emotion.

	Musicians			General Population (Butler and Kern, 2016)					
	*N*	Mean	*SD*	*N*	Mean	*SD*	*T*	*p*	Cohen’s *d*
Positive Emotion	601	7.06	1.50	31965	6.69	1.97	5.95	<0.0001	0.21
Engagement	601	7.33	1.34	31962	7.25	1.71	1.26	0.318	0.05
Relationships	601	7.25	1.70	31940	6.90	2.15	4.83	<0.0001	0.18
Meaning	601	7.64	1.58	31931	7.06	2.17	8.84	<0.0001	0.31
Accomplishment	601	7.32	1.38	31963	7.21	1.78	1.75	0.171	0.07
Overall Wellbeing	601	7.34	1.68	31966	7.02	1.66	5.04	<0.0001	0.20
Negative Emotion	601	4.04	1.76	31386	4.46	2.06	5.38	<0.0001	0.22

*All scales range from 0 to 10, where 0 = lowest and 10 = highest.*

While for general population samples the leading component of PERMA is *Engagement*, for musicians *Meaning* is the highest score. In fact, *Engagement* appears as the highest element for 10 out of the 11 validation sub-samples, when analyzed individually (Butler and Kern, 2016). For both musicians and the general population, *Positive Emotion* is the PERMA component with the lowest score. Musicians’ scores were significantly higher (*p* < 0.0001) than scores for general population for the sub-scales of *Positive Emotion*, *Relationships*, *Meaning* and for *Overall Wellbeing*, and significantly lower for *Negative Emotion* as determined by independent-samples t-tests conducted using summary values. Effect sizes were modest.

## Discussion

The results of this study suggest that the wellbeing profile of classical professional musicians, through the lens of the PERMA model, is largely positive. Indeed, not only do classical musicians score above the mid-point of the scale for all components of PERMA, they score significantly higher than general population indicators for three of the five elements: *Positive Emotion*, *Relationships*, and *Meaning* and, importantly, not below general population on *Engagement* and *Accomplishment*. Moreover, these high scores are generally transversal to all the types of music activity included in the study. Besides the evidence of good levels of positive functioning, scores on negative affect are also generally low and, importantly, slightly lower than general population indicators. Given our large sample size, when comparing with the general population sample scores, we are cautious in interpreting significance for such small mean differences as the ones we observed. Nevertheless, it is striking that musicians’ mean scores were not below those of general population for any of the PERMA components.

These results can seem surprising in relation to the extant literature on classical musicians’ health, as they apparently contradict the stereotype regarding the music profession as a high source of intense stressors and strains for wellbeing. Professional musicians appear to be more resilient toward these challenges than was once believed, perhaps preventing them from having a major impact on their self-reported wellbeing evaluations. These results bring to light yet another phenomenon permeating wellbeing research with musicians: the conceptual blurriness around wellbeing as a construct and the consequent methodological dangers it brings. Indeed, until now, the studies on musicians’ wellbeing have tended to target illbeing, and this may help to understand the results of the current study. If the formulations for wellbeing as *more than the absence of disorder* – shared by the World Health Organization and largely expanded by Positive Psychology research – are to be taken seriously, wellbeing assessment necessarily translates into measuring positive components of functioning alongside negative ones, and these may indeed represent different phenomena (Keyes, 2005). When looking closely at the body of research on classical musicians’ wellbeing thus far, assessment is almost exclusively done with recourse to measures of disorder (depression, stress, anxiety and social phobia, among others). As a consequence, conclusions can only be taken on illbeing, not wellbeing, and the scope for comparisons between studies remains limited.

Nevertheless, even when considering potential illbeing indicators – the *Negative Emotion* sub-scale – musicians stand well placed when looking at general population samples. In particular, given the centrality of MPA in previous research regarding musicians’ psychological health, the low score on the *Negative Emotion* subscale is, indeed, surprising. Anxiety in this study was assessed based on a single item and pointed toward general functioning, not performance-related situations; therefore, any interpretation needs to be extremely cautious. It is still noteworthy, however, to have such a low negative affect mean score, with a fairly small confidence interval, and that this mean score stands as significantly lower than that of the general population, even if the size of the effect is modest.

The role of *Meaning* for professional musicians’ wellbeing stands as one of our major findings. Previous qualitative accounts (Ascenso et al., 2017) had highlighted this as one of the key components structuring musicians’ wellbeing. The current study allowed for a look into quantitative indicators of meaning, placing them in context with the remaining components of wellbeing as defined by PERMA. This can help further understand the apparent dissonance between the mostly negative mental health profile of this professional group drawn in previous research (e.g., Steptoe, 1989; Parasuraman and Yasmin, 2000; Kenny et al., 2004) and the results of this study. Besides the conceptual diversity and the tendency for negative assessments already discussed, profiles of wellbeing based on affect alone will likely fail to fully grasp musicians’ experience of optimal functioning. A striking result in line with this argument refers to *Positive Emotion* as the lowest of all components of PERMA. Contentment and satisfaction alone may not be, therefore, good indicators of musicians’ wellbeing. Besides reinforcing how meaning remains a crucial cornerstone for classical musicians, our results also evidenced that musicians at a later stage of life report more meaning than younger musicians, in line with previous studies with general population samples (Steger et al., 2009).

Another result worth noting along with the higher *Meaning* for older musicians, is the concurrent decrease in *Negative Emotion*, and crucially, the weight of age in explaining the differences in the scores of these two scales when including Years of Experience in the analysis. The results seem to suggest that the challenges to wellbeing around the transition to professional phase reported in previous studies with musicians (MacNamara et al., 2008; Ascenso et al., 2017) depend more on the age of entry into the profession than on the number of years of experience *per se*. Naturally, older musicians will likely develop greater maturity that may help alleviate the reported transition challenges. Further research would benefit from including both variables and particularly, qualitative inquiry would help further understand this relationship in context.

As with most cross-sectional studies in musicians’ wellbeing, the question remains as to whether the differences associated with age are explained by the development of the positive components of functioning throughout the years or by *a priori* advantage of some musicians on these components, and concurrent drop-outs from the area of less psychologically fit professionals along the way. Previous qualitative accounts from eminent musicians (Ascenso et al., 2017) offered insight into this issue, pointing to the development of effective strategies toward wellbeing challenges with time. Retrospective accounts of the early years in the profession also reinforce this point, even by highly fit musicians. The two variables of age and years of experience, however, are not discriminated in previous accounts, and deserve to be explored further.

In tune with previous research (Steptoe and Fidler, 1987; Ascenso et al., 2017), the trend observed in our data for a decrease of *Negative Emotion* with age also brings back the question of whether the centrality of MPA is mainly experienced by younger musicians. Indeed, previous accounts suggest that older professionals do not consider MPA as a main challenge in maintaining wellbeing as a music performer, despite experiencing it (Ascenso et al., 2017). In a questionnaire on MPA, musicians might score high enough to be associated with a debilitating MPA experience; however, we argue that this is different from assuming that experiencing MPA equals low general psychological wellbeing. Not only do musicians develop strategies to cope (Ascenso et al., 2017), there are several other elements feeding into their evaluation of wellbeing as is evident from the results of this study. Interestingly, a recent study with music conservatoire students using the Short Warwick-Edinburgh Mental Wellbeing Scale (SWEMWBS) found higher wellbeing scores for this group when compared with similar age samples from previous studies (Araújo et al., 2017). The results call for a more extended assessment, given the brief nature of the scale, but highlight how the wellbeing experience of music students is perceived positively despite the high level of challenge.

Our results also provide indication that male and female musicians might be affected differently by the phase of transition to the profession, especially with regard to the experience of the overall sense of wellbeing and positive affect, two results that deserve further exploration. It would be particularly interesting to explore the role of normative developmental adaptations on the wellbeing of women musicians in the early career phase (such as motherhood, for example).

Regarding the dimension of positive *Relationships*, another central element of wellbeing within this group (Ascenso et al., 2017), there seems to be high satisfaction. Interestingly, no differences were found on this component for the different areas of musical activity, which included both musicians working mainly in collaborative settings and musicians with solo professional routines (soloists, composers and to some extent, conductors). Our previous qualitative inquiry had highlighted the challenge of social inclusion, in particular for solo musicians and in relation to social contexts outside the music world (Ascenso et al., 2017). It seems from the results of the current study that there is not a particular challenge around satisfaction with relationships across areas. It remains to be clarified if the participants’ interpretations of the items were primarily linked with an evaluation about work relationships or life outside of music. The items point to an overall evaluation, however, since participants were aware that this study was focused on musicians’ wellbeing, it is possible that the tendency of response was in relation to the work context. This clarification deserves attention in further research.

Finally, the results for *Engagement* and *Accomplishment* are also worth highlighting. Musicians’ experience of involvement in their tasks, the sense of handling their responsibilities effectively and achieving and progressing toward their goals, are not significantly different from the results of general population samples. This is an encouraging result given that nearly 40% of our sample consisted of professional orchestral musicians, a group previously associated with high levels of boredom at work (Parasuraman and Yasmin, 2000). The role of varying repertoire and music-making contexts in musicians’ engagement and sense of *Accomplishment* have been previously highlighted (Ascenso et al., 2017), in particular with orchestral musicians (Ascenso, 2016), and might help explain this result. All of the orchestras participating in this study maintain high dynamism around new music as well as community engagement projects, allowing for a wide diversity of experiences.

This study reveals several avenues for further research. The most urgent is the establishment of wellbeing assessments with musicians that are theoretically rooted, clearly operationalized and inclusive of the obvious multidimensionality and positive nature of the construct. This gap echoes a general challenge in psychology toward finding consensus on a universal definition of wellbeing. Furthermore, as Delle Fave and Kajan (2016) point out, there has been a tendency for work-related studies to be centered on office and factory employees and neglect professional activities such as the arts and crafts. Performing artists remain under-researched and can largely benefit from the most recent advancements in wellbeing assessment. In line with this, the relationship between the experience of positive functioning and psychological illness (the latter so widely documented in literature with musicians), deserves careful exploration. The description of negative or positive profiles have the danger of falling into a rather simplistic approach of just polling participants across discrete symptoms. We suggest that in further studies the experiences of wellbeing and illbeing of musicians be interpreted in relation to one another. Further understanding of the experience of meaning construction for musicians would be highly valuable as a follow-up from this study. Specifically, a profile of musicians’ experiences would benefit from integrating the recent conceptual refinement suggested by Martela and Steger (2016), acknowledging three different facets of meaning. Namely, *coherence* (a sense of comprehensibility and that one’s life makes sense), *purpose* (a sense of central aims and direction in life) and *significance* (a sense of life’s inherent value and having a life worth living) (Martela and Steger, 2016).

The pattern of lower average scores for women composers and conductors on *Positive Emotion*, *Relationships*, *Meaning* and *Overall Wellbeing* also deserves further attention. The high confidence intervals due to the small size of these sub-groups do not allow for a firm interpretation. However, the consistent pattern asks for further clarification with both groups, making use of a larger sample. Similarly, follow-up inquiry with male conductors, focusing on an in-depth understanding, in context, of the processes behind their consistently higher results across the same scales, particularly evident in the case of *Meaning*, would provide invaluable insight.

Extending this study to music students is also essential, especially considering the previous qualitative accounts on the particular challenges around the transition from student to professional life (Ascenso et al., 2017). High-profile music institutions such as orchestras and opera companies are becoming more engaged with promoting the inclusion and wellbeing of musicians in transition to the profession, with innovative initiatives which also deserve further attention (Ascenso et al., in press). A wellbeing profile with students would clarify possible routes for interventions with pre-professionals, aiming at a robust psychological preparation for professional life.

The replication of this study with musicians engaged in other musical styles (e.g., jazz and pop) would also represent a valuable addition to the literature, especially acknowledging the differences between these professional groups regarding freedom and autonomy in performance (Dobson, 2010). Furthermore, the growth of portfolio careers in music in recent years makes the rigid categories of professional activity in this context rather spurious. A large number of professional musicians are engaged both in performance and teaching. The explicit inclusion of portfolio musicians would therefore be another much-needed route for further investigation.

Given the prevalence of physical challenges so well documented across all phases of musicians’ careers, further study is needed on the relationship between physical and mental health for this group, in particular acknowledging previous results linking propensity for injury for musicians experiencing psychological distress (Baadjou et al., 2016).

Research on specific professional groups helps lay the groundwork for comprehensive and tailor-made interventions. The PERMA model stands as a useful framework at a practical level to incorporate in educational and professional settings, in the endeavor of promoting professional classical musicians’ wellbeing across different dimensions. It seems timely to create wellbeing initiatives for musicians that go beyond minimizing performance anxiety or preventing physical injury and that take steps toward the deliberate promotion of positive functioning. Another main thread for further research is, therefore, the development of empirical support and optimization for such positive interventions within the sector.

Finally, our investigation does not permit conclusions based on causality. A prospective cohort study would be needed to explore further how the observed differences can be explained. Additionally, the limitation of the self-selection bias that our procedure entailed would ideally be considered in further studies, allowing for stronger claims on generalization.

This study has presented a cross-sectional wellbeing profile of professional classical musicians, pointing to high levels of wellbeing as defined by the PERMA model. It has shed light on what components are most conducive to flourishing for this professional group, revealing the centrality of *Meaning*. We argue, therefore, that a re-direction toward positive multidimensional assessment of wellbeing, theory-driven research and the awareness of the inherent idiosyncratic elements of optimal functioning in the case of musicians stand as necessary next steps in the pursuit of better approaches toward mental health promotion among this professional group.

## Author Contributions

SA conducted the study and wrote the manuscript. All authors contributed to the study design, interpretation of results, and proofreading.

## Conflict of Interest Statement

The authors declare that the research was conducted in the absence of any commercial or financial relationships that could be construed as a potential conflict of interest.
